# Collagen-Based Microspheres for Biomedical Applications in Drug Delivery and Tissue Engineering

**DOI:** 10.3390/biomimetics11040233

**Published:** 2026-04-01

**Authors:** Mohammad Jahir Raihan, Zhong Hu, Solaiman Tarafder

**Affiliations:** 1 Biomaterials and Mechanobiology Laboratory, Department of Mechanical Engineering, Jerome J. Lohr College of Engineering, South Dakota State University, Brookings, SD 57007, USA; 2 Department of Mechanical Engineering, Jerome J. Lohr College of Engineering, South Dakota State University, Brookings, SD 57007, USA

**Keywords:** collagen, microspheres, immunogenicity, microfluidics, regenerative medicine, 3D bioprinting

## Abstract

Collagen, the most abundant extracellular matrix (ECM) protein, has emerged as a cornerstone biomaterial in drug delivery and regenerative medicine due to its intrinsic biocompatibility, biodegradability, and low immunogenicity. Engineering collagen into microspheres transforms its functionality beyond bulk scaffolds by increasing surface area, enabling minimally invasive delivery, and providing precise control over degradation, mechanical properties, and therapeutic release. This review provides a comprehensive analysis of collagen-based microspheres, with a particular focus on their dual role as biomimetic microenvironments and delivery systems. Recent advances in fabrication strategies, including emulsification, microfluidics, spray-drying, and electrospraying, are discussed in the context of scalability, size control, and payload encapsulation. Composite approaches that incorporate bioactive minerals, polysaccharides, or synthetic polymers are highlighted for their ability to enhance mechanical performance and biological function. We further examine characterization frameworks that link microscale structure and physicochemical properties to biological outcomes, with emphasis on how collagen microspheres replicate key structural, mechanical, and signaling features of native tissue microenvironments. Collagen microspheres have demonstrated broad utility as controlled delivery platforms, cell-instructive microcarriers, and injectable systems for tissue regeneration, including applications in bone, cartilage, skin, and nerve repair, as well as advanced wound care and localized cancer therapy. Finally, we critically assess current challenges related to scalable manufacturing, sterilization compatibility, and batch reproducibility, and outline emerging solutions such as recombinant collagen, advanced biofabrication, and stimuli-responsive systems. Collectively, collagen microspheres represent a powerful and adaptable platform poised to advance next-generation regenerative and therapeutic technologies.

## 1. Introduction

Collagen, the most abundant protein in the mammalian extracellular matrix (ECM), has long been a cornerstone of biomaterials research due to its essential role in maintaining structural integrity in tissues such as skin, bone, tendon, and cartilage [1,2,3]. Its intrinsic bioactivity, including cell-adhesive motifs and enzymatic degradability, makes it particularly attractive for constructing scaffolds that aim to recapitulate native tissue environments. Despite these advantages, traditional bulk collagen constructs, such as hydrogels and sheets, present several limitations [4]. These include difficulty in conforming to irregular defect geometries, limited suitability for minimally invasive delivery, and insufficient control over the spatial and temporal presentation of therapeutic signals [5,6,7]. In addition, the rapid enzymatic degradation of non-crosslinked bulk collagen can compromise mechanical integrity and long-term function in vivo [8,9]. More fundamentally, bulk collagen systems often act as static ECM analogs. Although they reproduce collagen as a material component, they do not readily capture the microscale heterogeneity, spatial organization, or dynamic remodeling that characterize native tissue microenvironments [10,11,12].

Collagen-based microspheres have emerged as a versatile platform that addresses many of these limitations by transitioning from monolithic scaffolds to modular particulate systems [13]. Their high surface area-to-volume ratio enhances cell material interactions and improves nutrient and oxygen transport, which is particularly beneficial for cell encapsulation and tissue regeneration applications [14,15]. Their microscale size enables injectable delivery, allowing access to deep or irregular tissue defects while reducing surgical invasiveness and associated complications [16,17]. In addition to these practical advantages, the particulate nature of microspheres enables localized and modular presentation of matrix components, which is difficult to achieve in bulk constructs and is critical for mimicking the spatial organization of native ECM [18,19].

A major strength of collagen microspheres lies in their ability to function as controlled delivery systems. By tuning microsphere size, porosity, and crosslinking density, it is possible to regulate the release of encapsulated drugs, growth factors, or genetic material [20,21,22]. This level of control is essential because tissue repair and regeneration are governed by temporally coordinated signaling events rather than static biochemical conditions [23]. Compared with bulk materials, which often exhibit rapid burst release, microspheres can be engineered to provide sustained and localized delivery profiles that better align with physiological healing processes [23,24]. As a result, collagen microspheres serve not only as carriers of therapeutic agents, but also as systems that enable precise spatial and temporal control of bioactive signals. Beyond their role as delivery vehicles, collagen microspheres are increasingly recognized as biomimetic microenvironments that can replicate key features of native ECM. Structurally, the fibrillar collagen network within microspheres provides a three-dimensional architecture that supports cell attachment, spreading, and migration through natural cell matrix interactions [25,26].

The microscale and modular nature of collagen microspheres enable better representation of the spatial complexity of native tissues. Unlike uniform bulk scaffolds, they can be assembled into heterogeneous environments with varied composition, properties, and payloads, allowing formation of localized niches that more closely resemble native tissue organization [27,28]. This feature is particularly important for applications requiring zonal or gradient structures, such as osteochondral and tendon-to-bone interfaces. Mechanically, collagen microspheres offer tunable properties that regulate cell behavior through mechanosensitive pathways. Although collagen is inherently weaker than many synthetic polymers, its stiffness can be adjusted through crosslinking or composite design to match tissue-specific requirements while maintaining biological activity [17,29,30]. In contrast, synthetic systems often provide precise mechanical control but lack intrinsic bioactivity, limiting their ability to support dynamic cell matrix interactions without additional modification [31].

Their signaling capability further enhances their biomimetic function. Collagen undergoes enzymatic degradation and cell-mediated remodeling, enabling dynamic reciprocity between cells and the matrix, which is essential for tissue development and healing. Such interactions are difficult to achieve in inert or nondegradable materials. In addition, collagen microspheres can deliver therapeutic molecules alongside intrinsic matrix cues, providing both structural and signaling functions. This versatility can be further expanded through composite design [29]. Incorporation of materials such as alginate, chitosan, or hydroxyapatite (HAp) improves mechanical properties, controls degradation, and introduces additional functionalities, including osteoconductivity and antibacterial effects [17,30,32]. These strategies allow collagen microspheres to better reflect the compositional complexity of native tissues [27,28]. Advances in fabrication techniques have also improved their design and reproducibility. While conventional water-in-oil (W/O) emulsification remains widely used, more precise methods such as microfluidics and electrospraying enable tight control over size, shape, and uniformity [33]. Comprehensive characterization of morphology, mechanical properties, degradation, and biological performance is essential, with increasing emphasis on linking microscale structures to functional outcomes relevant to tissue behavior [32,34].

Collagen-based microspheres have been widely applied in drug delivery, tissue engineering, wound healing, and biofabrication [35]. In regenerative medicine, they function as injectable scaffolds for delivering cells and therapeutic factors to repair bone, cartilage, neural, and other tissues [34,35]. In wound healing, they enable localized delivery of antibiotics or growth factors [35,36]. Their integration with three-dimensional (3D) bioprinting has enabled microsphere-based bioinks that improve spatial control over cell distribution and matrix composition. This is critical for constructing heterogeneous tissues with region-specific functionality [37]. Advances in biofabrication have also enabled the production of collagen microspheres with improved uniformity and scalability, supporting their translational potential [38]. Collectively, these developments position collagen microspheres as both effective delivery systems and modular building blocks for bottom-up tissue assembly and patient-specific therapies [39].

Despite substantial progress, several challenges remain. Achieving predictable long-term stability and controlled degradation in vivo continues to be a key concern [40]. Sterilization methods must be carefully optimized to preserve structural and biological integrity, particularly for systems that incorporate sensitive biomolecules or living cells [27]. In addition, producing large amounts of uniform microspheres is still technically and economically challenging, especially with advanced fabrication methods [41,42]. Addressing these issues will be essential for broader clinical adoption [43].

The objective of this review is to provide a comprehensive overview of collagen-based microspheres, focusing on their role as biomimetic microenvironments and delivery systems. It explains how these systems mimic the structural, mechanical, and biological features of the native extracellular matrix while enabling controlled therapeutic delivery. Furthermore, it covers advances in fabrication, characterization, and biomedical applications, and discusses current challenges and future opportunities. Overall, this review serves as a valuable resource for researchers and clinicians seeking to understand and leverage the unique potential of collagen-based microspheres for future biomedical applications.

## 2. Collagen: Sources, Structure, and Properties

Collagen is the most abundant structural protein in the mammalian extracellular matrix (ECM) and remains one of the most widely used natural polymers in biomedical research and clinical applications [44]. Its ubiquity in native tissues, combined with intrinsic biocompatibility, enzymatic degradability, and the presence of integrin-binding motifs, makes it a particularly attractive base material for microsphere systems [45,46]. However, not all collagens are equivalent. The source of the protein, its molecular architecture, and any subsequent chemical or physical modifications collectively determine how collagen behaves during microsphere fabrication and after implantation in vivo [47]. A clear understanding of these factors is therefore essential for rational design and successful translation of collagen-based microspheres for biomedical applications [48,49].

### 2.1. Sources of Collagen

**Figure 1:**
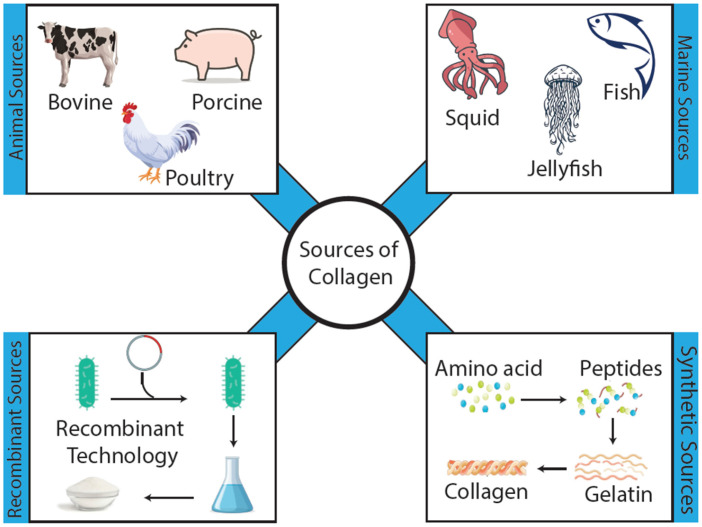
**Major sources of collagen for biomedical applications.** Collagen can be derived from multiple sources, including animal tissues such as bovine, porcine, and poultry, as well as marine organisms such as fish, squid, and jellyfish. It can also be produced using recombinant systems with engineered cells and synthetic approaches involving amino acid and peptide assembly. Different sources of collagen offer variations in biochemical properties, immunogenicity, and scalability, supporting its broad use in drug delivery, tissue engineering, and regenerative medicine.

Collagen for biomedical applications is derived from both animal and non-animal sources, each offering distinct advantages and limitations in terms of purity, safety, immunogenicity, and regulatory considerations [48,50,51]. As illustrated in Figure 1, major sources include mammalian, marine, recombinant, and synthetic systems. Historically, animal tissues have dominated due to their availability and relatively low cost, while recombinant technologies have recently emerged as promising alternatives to address concerns related to variability and disease transmission [52].

Mammalian tissues, particularly bovine and porcine skin, tendons, and bones, remain the most commonly used industrial sources of type I collagen [53]. Acidic or enzymatic extraction methods solubilize fibrillar collagen while preserving much of its triple-helical structure, yielding high quantities of protein suitable for forming gels, sponges, and microspheres [54]. Bovine collagen has been extensively used in hemostatic agents, dermal fillers, and tissue scaffolds due to its mechanical strength and close structural similarity to human collagen [55,56]. However, the use of bovine and porcine sources presents potential drawbacks, including xenogeneic antigenicity, batch variability, and the risk of zoonotic disease transmission such as bovine spongiform encephalopathy [57,58]. In addition, cultural and religious considerations may limit the acceptance of these materials in certain populations [59]. From a functional standpoint, mammalian collagen is often considered the benchmark because its relatively high thermal stability and robust fibrillar assembly support stronger matrices and slower degradation compared to many alternative sources [60].

Marine-derived collagen has gained increasing attention as a safer and more broadly acceptable alternative [61]. It can be extracted from fish skin, scales, bones, swim bladders, and invertebrates such as jellyfish and sponges [62]. Although marine collagen is predominantly type I, it typically exhibits lower denaturation temperatures and distinct amino acid compositions compared to mammalian collagen, reflecting adaptation to colder environments [63]. These features can be advantageous for processing at mild temperatures and for applications requiring faster degradation. However, they may also necessitate careful control during microsphere fabrication to prevent premature denaturation [62]. Importantly, marine collagen largely avoids concerns related to mammalian disease transmission and is more acceptable across diverse cultural contexts. At the same time, species-dependent variability in molecular weight, crosslink density, and thermal stability requires careful characterization and process control [64,65]. Compared to mammalian collagen, marine collagen often forms less mechanically robust networks and degrades more rapidly under physiological conditions unless reinforced through crosslinking or composite strategies [64]. As a result, it is particularly suitable for soft tissue or rapidly remodeling applications but may be less appropriate for applications requiring prolonged mechanical stability [66].

Beyond animal-derived sources, recombinant collagen and collagen mimetic systems provide a pathway toward well-defined and human-like materials. Recombinant human collagen can be produced using yeast, bacterial, plant, or mammalian expression systems, enabling precise control over amino acid sequence, post-translational modifications, and incorporation (or removal) of specific bioactive motifs [67,68]. These systems reduce immunogenicity, eliminate animal-derived contaminants, and improve batch consistency, which is especially important for regulated biomedical products. However, production complexity and cost remain significant challenges, and large-scale manufacturing infrastructure is still evolving. In parallel, synthetic collagen mimetic peptides and peptide polymer hybrids have been developed to replicate key structural and functional features of native collagen while allowing controlled synthesis and modular functionalization [68,69]. Although these systems are not yet as widely used as native collagen in microsphere applications, they represent an important direction for developing highly controlled and customizable biomaterials.

Compared to mammalian collagen, recombinant systems offer advantages in compositional control, safety, and reproducibility, rather than inherently superior mechanical performance [70,71]. Their fibrillogenesis, mechanical properties, and degradation behavior depend strongly on the expression system and subsequent processing strategies, including crosslinking and blending [72,73]. Consequently, mammalian collagen remains advantageous when robust fibrillar assembly and mechanical strength are required, whereas recombinant collagen is particularly attractive when precise molecular control and reduced biological variability are critical for translation [74,75].

Overall, collagen sources should be considered a structure–property–function variable rather than a simple material choice. Mammalian collagen typically provides stronger fibrillar assembly and slower degradation. Marine collagen offers broader acceptability with faster remodeling but lower stability, and recombinant collagen enables precise compositional control with improved reproducibility. For collagen microspheres, these source-dependent differences directly influence fabrication conditions, particle integrity, degradation kinetics, and in vivo performance [75,76].

### 2.2. Collagen Structure and Properties

**Figure 2:**
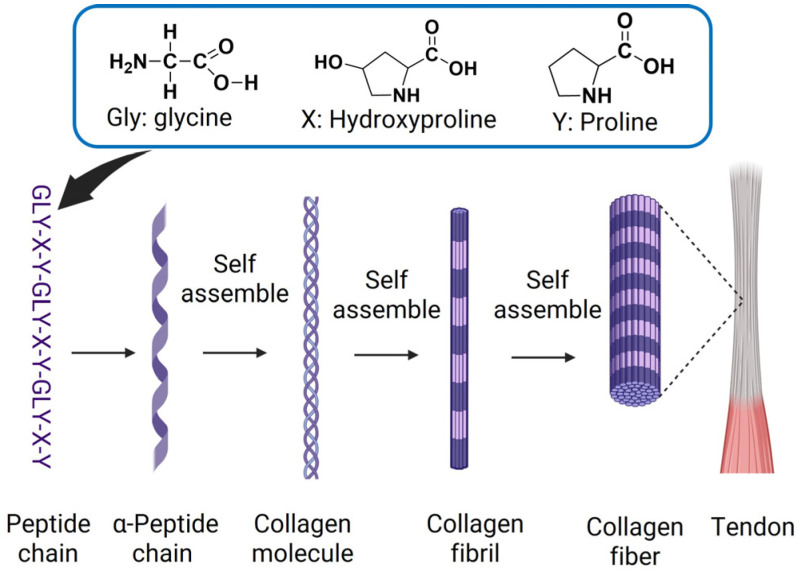
**Hierarchical structure and self-assembly of collagen.** Collagen is built from a repeating Gly–X–Y sequence that forms an α chain. These chains self-assemble into a triple-helical procollagen molecule. After procollagen peptidase removes the propeptides, mature collagen molecules assemble into fibrils. These fibrils then bundle into larger fibers, forming the hierarchical structure that governs the architecture and mechanical behavior of collagen-rich tissues.

Collagen’s utility as a biomaterial comes from its hierarchical structure. Each molecule consists of three α-chains rich in Gly-X-Y repeats, where X and Y are often proline and 4 hydroxyproline. Glycine enables tight packing within the triple helix, while proline and hydroxyproline stabilize the rigid conformation. This multiscale assembly process is illustrated schematically in Figure 2, beginning from the Gly-X-Y peptide sequence to α-chain formation, followed by self-assembly into a triple-helical procollagen molecule. Subsequent processing by procollagen peptidase leads to collagen formation, which then self-assembles into collagen fibrils and fibers. The collagen monomer consists of a long central Gly-X-Y sequence with N and C terminal regions on either side and undergoes a well-established pathway of intracellular maturation and triple helix formation [77].

After synthesis, procollagen is secreted into the extracellular space, where it undergoes enzymatic processing to form collagen. Following hydroxylation and limited glycosylation, the α chains assemble into tropocollagen, a right-handed triple helix stabilized by interchain hydrogen bonding and bound water, which contributes to its thermal and proteolytic stability [78,79]. Tropocollagen molecules further assemble into fibrils characterized by a 67 nm D band periodicity, which bundles into fibers and fascicles. This hierarchical organization provides high tensile strength and controlled viscoelastic behavior. Reconstituted collagen, such as neutralized and thermally induced gels, can form similar fibrillar networks that govern the mechanical properties of hydrogels and microspheres [80].

Collagen is biodegradable through the enzymatic activity of matrix metalloproteinases, cathepsins, and other proteases, allowing microspheres to be remodeled and gradually replaced by native ECM. Degradation rates can be tuned by adjusting fibril density, crosslinking, or incorporation of copolymers, which is particularly important for controlling drug release kinetics [81,82,83]. As a native ECM component, collagen is inherently biocompatible and supports cell adhesion through integrin-binding motifs such as Gly-Phe-Hyp-Gly-Glu-Arg (GFOGER)- and Arg-Gly-Asp (RGD)-like sequences [84]. In microsphere systems, these motifs facilitate direct cell matrix interactions. However, the presence of telopeptides and residual impurities can contribute to immunogenicity. Therefore, medical grade collagen is typically purified or partially processed to reduce antigenicity while maintaining bioactivity [85].

These structural and biochemical features are central to the biomimetic function of collagen microspheres. Collagen naturally provides a fibrillar architecture, cell-adhesive ligands, hydrated three-dimensional organization, and protease sensitive remodeling behavior, enabling it to reproduce several key features of native ECM more effectively than many inert polymer systems [86,87]. At the same time, source-dependent variations in molecular composition and thermal stability, as discussed in Section 2.1, influence how well these ECM-like properties are preserved during extraction, processing, and microsphere fabrication [88]. For example, collagen sources that retain triple-helical stability and fibrillar assembly tend to produce microspheres with greater structural integrity, slower degradation, and more sustained presentation of biochemical cues. In contrast, less stable collagen may favor faster remodeling but reduced mechanical persistence [89,90]. Accordingly, collagen source and molecular structure should be considered together, as both govern the performance of collagen microspheres, including particle stability, degradation kinetics, cell matrix signaling, and the extent to which the system replicates native tissue microenvironments [91].

## 3. Preparation and Fabrication Techniques for Collagen-Based Microspheres

The transition from bulk collagen gels or sponges to microscale particulates requires fabrication techniques that can produce discrete, generally spherical structures while preserving collagen’s bioactivity. At the same time, these methods must allow control over particle size, morphology, porosity, and encapsulation of therapeutic agents or cells. A range of approaches has been developed, from conventional emulsification to more advanced microfluidic and electrospraying strategies, each with distinct advantages and tradeoffs in terms of scalability, uniformity, and processing conditions. Different techniques with principle, size controls, advantages, and their limitations are summarized in Table 1.

### 3.1. Emulsification Methods

**Figure 3:**
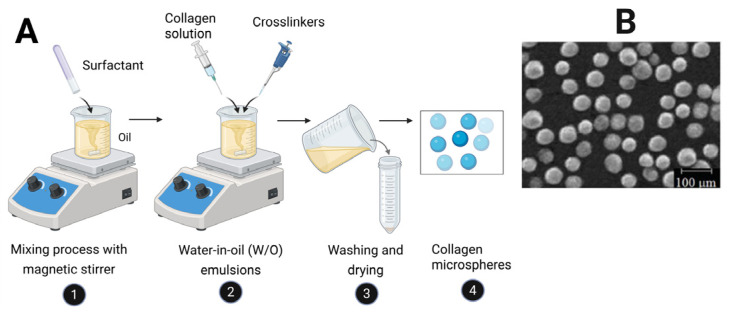
**Collagen microsphere fabrication by water-in-oil emulsion and application for drug delivery.** Collagen microspheres prepared using water-in-oil emulsion (**A**) and the prepared microsphere for the sustained release of steroidal saponins (**B**) [33].

Water-in-oil (W/O) emulsification is one of the earliest and most widely used methods for fabricating collagen microspheres due to its simplicity, versatility, and scalability. In this approach, an aqueous collagen phase is dispersed into an immiscible oil phase under mechanical agitation, forming collagen-rich droplets that are subsequently stabilized into microspheres [109,110]. Although conceptually straightforward, droplet formation and stabilization are governed by interfacial phenomena, fluid shear, and collagen gelation or crosslinking mechanisms. As illustrated in Figure 3, the process involves preparation of a surfactant-containing oil phase, dispersion of the aqueous collagen phase under stirring, stabilization of droplets, and recovery of microspheres through phase separation, washing, and drying [13,92].

Stable droplet formation requires reduction in interfacial tension between the aqueous and oil phases, typically achieved using surfactants such as Span 80 or Tween 80 [23,111,112]. These surfactants adsorb at the interface and reduce coalescence, enabling formation of smaller and more stable droplets. Surfactant concentration influences droplet size and stability, although excessive amounts may complicate purification or affect cytocompatibility [113,114]. The oil phase also contributes through its viscosity and density, with common choices including mineral oil, liquid paraffin, olive oil, and hexane, each affecting droplet stability and separation behavior. Thus, interfacial stabilization plays a central role in emulsion formation and reproducibility.

Droplet formation is driven by mechanical shear, where hydrodynamic forces overcome interfacial tension to generate discrete droplets. Particle size is primarily controlled by agitation rate and surfactant concentration, with higher agitation producing smaller droplets and lower speeds yielding larger particles with broader size distributions [115]. Additional factors such as phase viscosity, aqueous-to-oil ratio, collagen concentration, and temperature further influence droplet formation. In particular, increasing collagen concentration raises viscosity and promotes larger particle formation, while temperature must be carefully controlled to prevent premature fibrillogenesis.

Following droplet formation, stabilization converts the droplets into solid or gel-like microspheres. In collagen systems, this is achieved through thermal fibrillogenesis, ionic interactions, or chemical crosslinking. Temperature-induced fibrillogenesis allows neutralized collagen to self-assemble into fibrils at physiological conditions, forming a stable internal network [94]. Chemical crosslinking, especially using carbodiimide systems such as 1-ethyl-3-(3-dimethylaminopropyl)carbodiimide hydrochloride (EDC) and N-hydroxysuccinimide (NHS), is widely employed to enhance structural integrity, reduce swelling, and control degradation by forming covalent bonds between carboxyl and amine groups without leaving residual linkers [23,116]. Typically, microspheres are stabilized, recovered through ethanol-assisted phase separation, and washed repeatedly to remove residual oil, surfactant, and reagents.

More complex formulations can be achieved using composite systems and alternative crosslinking strategies. For example, collagen chitosan microspheres can be formed by dissolving each component in dilute acetic acid, mixing them into an aqueous phase, and dispersing into a surfactant stabilized oil phase, followed by glutaraldehyde crosslinking and subsequent washing and drying [17]. Although effective, some crosslinkers such as glutaraldehyde require extensive washing due to potential toxicity [23,93]. Similarly, collagen–gellan gum-β tricalcium phosphate (β-TCP) microspheres can be stabilized using a combination of ionic gelation with CaCl_2_ and chemical crosslinking with EDC [93].

In addition, double emulsion systems such as water-in-oil-in-water (W/O/W) introduce an additional aqueous phase to improve encapsulation of sensitive payloads, followed by crosslinking, solvent separation, washing, and drying [13,23,117]. Patent-based approaches further enhance emulsification through rapid droplet generation, controlled setting, crosslinking, and downstream processing, highlighting ongoing efforts to improve scalability and reproducibility [92]. After stabilization, microspheres are recovered by breaking the emulsion using solvents such as ethanol, followed by centrifugation and repeated washing to remove residual components [13,23]. Depending on the application, microspheres may be used in hydrated form, vacuum dried, or lyophilized. Postfabrication loading of drugs or growth factors can also be achieved through diffusion-based methods, such as soaking microspheres in bioactive solutions [33].

Overall, emulsification remains a foundational technique for collagen microsphere fabrication due to its flexibility, scalability, and compatibility with a wide range of formulations. Mechanistically, it involves three key steps: reduction in interfacial tension by surfactants, shear-driven droplet formation, and stabilization through gelation or crosslinking. Its main advantage is accessibility and adaptability, while its primary limitation is the relatively broad particle size distribution associated with batch processing. Nevertheless, it remains highly relevant, particularly for applications where simplicity and large-scale production are prioritized.

### 3.2. Microfluidics

Microfluidic fabrication has emerged as a highly controlled alternative to conventional emulsification for producing collagen microspheres with narrow size distributions and reproducible structure [99,118]. Unlike bulk emulsification, where droplet formation occurs under turbulent and statistically variable conditions, microfluidic systems confine fluid flow within micron-scale channels, where droplet generation is governed by well-defined hydrodynamic and interfacial forces [119]. This level of control makes microfluidics particularly attractive for applications such as controlled release, cell encapsulation, and tissue engineering, where particle uniformity strongly influences performance [120,121].

In droplet-based microfluidics, an aqueous collagen phase, optionally containing cells or bioactive payloads, is introduced as the dispersed phase, while an immiscible fluid serves as the continuous phase [118]. At geometries such as T junctions or flow-focusing regions, the continuous phase compresses and elongates the dispersed phase, leading to controlled necking and pinching off into uniform droplets [122]. This process, often described as hydrodynamic focusing, results in periodic and highly reproducible droplet formation due to the confined and laminar flow environment [123].

Droplet formation is governed by the balance between viscous forces and interfacial tension, commonly described by the capillary number. When viscous forces dominate, the collagen stream is more readily deformed, resulting in smaller droplets. Accordingly, droplet size can be precisely tuned by adjusting flow rates, fluid viscosities, and channel geometry [124]. In practice, increasing the continuous phase flow rate enhances shear and reduces droplet size, whereas increasing the dispersed phase flow rate produces larger droplets. Because collagen viscosity varies with concentration, pH, and temperature, these parameters must be optimized alongside flow conditions to achieve consistent particle size and structure [124].

A key advantage of microfluidics is the prevention of droplet coalescence [118]. In contrast to bulk emulsification, where droplets frequently collide and merge, microfluidic systems generate droplets sequentially and transport them in an ordered laminar flow, minimizing interactions. The presence of surfactants further stabilizes droplets by reducing interfacial tension. In addition, rapid downstream gelation or crosslinking can be used to fix droplet structure immediately after formation [125]. For collagen, temperature-induced fibrillogenesis is commonly employed, where droplets are exposed to physiological temperatures to form stable fibrillar networks [118].

**Figure 4:**
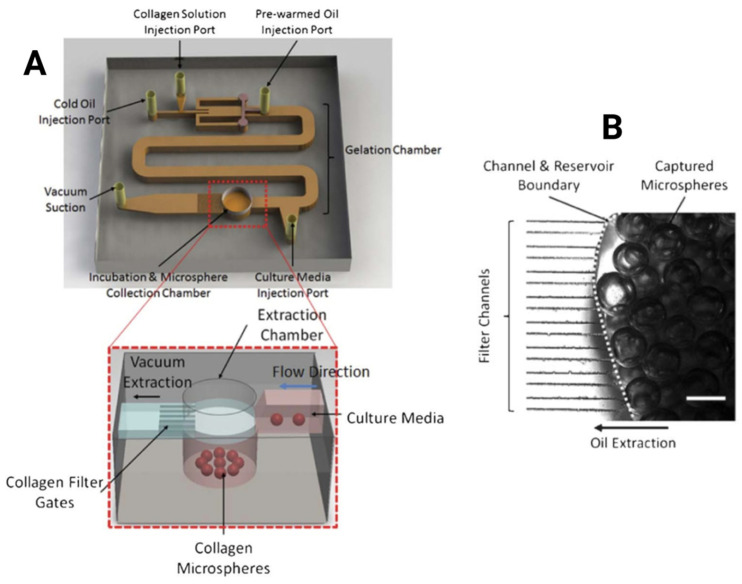
**Microfluidic chip design for collagen microsphere preparation.** (**A**) Schematic of a chip comprising three modules—droplet formation, gelation, and extraction—with an enlarged view of the extraction chamber (red dashed box) [96]. (**B**) Representative microfluidic workflow showing formation of collagen droplets at the junction, their downstream gelation, and subsequent transfer/extraction into aqueous medium to obtain uniform collagen microspheres [96].

Advanced lab-on-chip systems integrate multiple steps, including droplet generation, gelation, and extraction, within a single device [126]. In collagen-specific platforms, droplets are formed at a microfluidic junction between a chilled collagen or collagen suspension and a surfactant-containing oil phase as shown in Figure 4. These droplets are rapidly gelled by heating, which prevents coalescence and maintains size uniformity [96]. Some systems also include extraction chambers or phase transfer zones to transfer microspheres into aqueous media. This improves recovery efficiency and cell viability compared with centrifugation-based methods.

Microfluidics also enables the fabrication of more complex architectures [127]. Composite and engineered microcarriers can be produced by combining emulsion formulations with controlled droplet generation and solidification [118]. For example, collagen-patterned poly(lactic-co-glycolic acid) (PLGA) microcarriers can be fabricated using two-phase microfluidic systems, yielding highly uniform particles suitable for cell expansion and differentiation studies [95]. More broadly, coaxial and multiphase microfluidic designs enable the production of core shell or multilayer microspheres with high encapsulation efficiency under mild processing conditions [128].

A defining feature of microfluidic fabrication is monodispersity. Because droplet formation occurs under stable laminar flow and fixed geometries, nearly identical particles can be generated over extended periods [129]. This uniformity results in consistent diffusion pathways, predictable drug release, and reproducible cell loading, which are difficult to achieve with conventional batch emulsification [118]. It also improves quality control and facilitates mechanistic comparisons across studies. Despite these advantages, microfluidics remains limited for large-scale manufacturing due to inherently low throughput, susceptibility to channel clogging, and reliance on precision fabricated devices. Microfluidic systems typically operate at low throughput, and large-scale production requires parallelization of multiple channels or continuous flow designs while maintaining process stability and uniformity [99,130]. Parallelization strategies, such as multiplexed microfluidic chips, can increase production rates, but they introduce additional complexity in flow control, device fabrication, and quality assurance, thereby increasing capital and operational costs. Moreover, maintaining consistent droplet formation across multiple channels requires tight regulation of pressure, viscosity, and interfacial properties, which become increasingly challenging at scale.

Overall, microfluidics provides a high-precision approach for fabricating collagen microspheres through controlled droplet generation, minimized coalescence, and integrated processing. Its ability to produce highly uniform particles with tunable size and complex architectures makes it particularly valuable for advanced biomedical applications, although challenges in scaling remain to be addressed.

### 3.3. Spray-Drying

**Figure 5:**
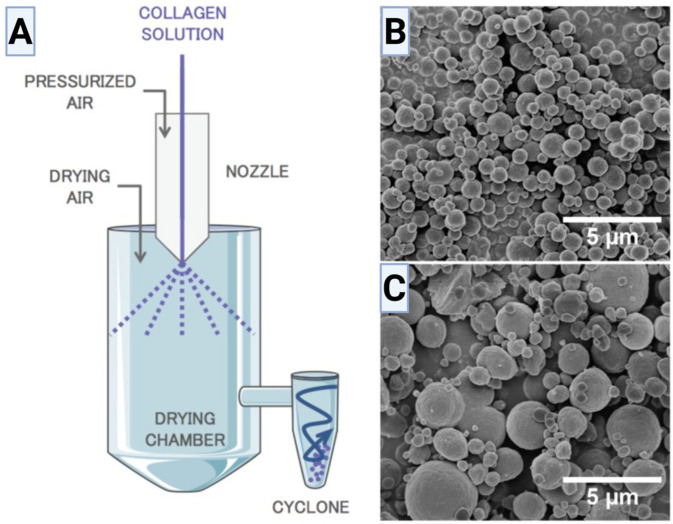
**Spray-drying-based fabrication of collagen microbeads:** (**A**) Schematic of the preparation of collagen microbeads from collagen solution. (**B**,**C**) SEM imaging of collagen microbeads produced by spray-drying collagen solutions [104].

Spray-drying is a scalable and industrially established technique for producing collagen-based microparticles through rapid drying of atomized droplets. Unlike emulsification and microfluidics, which generate droplets within an immiscible liquid phase, spray-drying forms droplets in a gas environment and converts them directly into dry particles through solvent evaporation. This method is particularly attractive for producing dry, storage-stable collagen particles that can later be rehydrated or incorporated into composite biomaterial systems [131]. As illustrated in Figure 5A, the process involves atomization through a nozzle using pressurized air, drying within a heated chamber, and particle collection via a cyclone. The droplet-to-particle transition occurs on short timescales, and the resulting morphology depends strongly on formulation and drying conditions [104].

The process begins with atomization of a collagen solution or dispersion into fine droplets. Droplet size is influenced by nozzle geometry, atomization pressure, feed viscosity, and flow rate. Smaller droplets dry more rapidly and typically yield smaller particles, whereas larger droplets require longer residence times and may produce broader size distributions. Collagen feed concentration plays a critical role, as dilute solutions may form low-mass or collapsed particles, while higher concentrations increase viscosity and produce larger or structurally distinct particles. As shown in Figure 5B,C [104], an increase in collagen concentration (e.g., 0.7 versus 5.0 mg mL^−1^) significantly affects particle size and morphology [104,132].

After atomization, droplets enter the drying chamber where solvent evaporation begins immediately. Evaporation first occurs at the droplet surface, leading to accumulation of collagen and dissolved components at the interface. As drying proceeds, a solid or semi-solid shell may form, and the rate of shell formation is governed by drying temperature, solvent volatility, airflow, droplet size, and solute concentration [133]. If evaporation is rapid relative to internal diffusion, hollow or wrinkled particles may form, whereas slower drying produces denser and more uniform structures. Thus, particle morphology is determined not only by initial droplet size but also by solvent removal kinetics [134].

In practice, particle size and morphology are controlled by inlet and outlet temperatures, atomization conditions, airflow, and feed rate. For example, typical operating conditions include feed rates around 15 mL per minute, inlet temperatures near 110 °C, outlet temperatures around 85 °C, atomization pressures of approximately 4 bar, and airflow rates of about 35 m^3^ per hour [103]. General trends from polymer microsphere systems indicate that larger nozzle diameters and higher feed rates increase particle size, whereas higher atomizing air input reduces particle size. A key operational constraint is avoiding incomplete solvent removal, which can occur at low inlet temperatures or high feed rates in aqueous systems [102]. For collagen-based systems, process parameters must also be optimized to minimize thermal denaturation of the triple helix and preserve the activity of encapsulated biomolecules. Careful selection of temperature, residence time, and feed composition is therefore essential to balance drying efficiency with protein stability [104].

Spray-drying also enables the fabrication of collagen inorganic composite systems. For example, HAp microspheres can be produced by spray-drying an aged slurry followed by calcination and then incorporated into collagen matrices for scaffold fabrication, such as through freeze-drying [101]. These hybrid systems combine the osteoconductivity of HAp with the biological functionality of collagen, making them particularly relevant for bone tissue engineering. In addition, internal particle architecture can be tailored during spray-drying using gas-forming additives. Hollow or porous microspheres can be generated using porogens such as ammonium bicarbonate, typically at concentrations of 1 to 7 wt.%, with drying conditions around 170 °C inlet and 100 °C outlet temperatures followed by post-treatment [100,135]. These structures increase the surface area and enhance mass transport and cell interactions, although careful optimization is required to prevent structural damage due to thermal and interfacial stresses.

Overall, spray-drying produces collagen microparticles through atomization and rapid solvent evaporation, offering scalability, continuous operation, and dry, storage-stable products. However, thermal exposure can partially denature collagen and reduce bioactivity, particularly for protein- or growth factor-loaded systems. In addition, shear and interfacial stresses during atomization may affect structural integrity, while rapid drying can lead to heterogeneous particle morphology and variable porosity. Process outcomes are highly sensitive to parameters such as temperature, feed composition, and atomization conditions, making reproducibility challenging [136]. Furthermore, the method is generally unsuitable for cell-laden systems and may require stabilizing additives, which introduce additional complexity and regulatory considerations.

### 3.4. Electrospraying

Electrospraying, also known as electrohydrodynamic spraying, is a particle fabrication method that uses an electric field rather than mechanical shear or thermal drying to generate droplets [137]. It is particularly attractive for collagen and collagen composite microspheres because it enables particle formation under relatively mild thermal conditions and avoids the need for a bulk oil phase [108]. In this process, a polymer solution is delivered through a metallic needle subjected to a high voltage relative to a grounded collector. The electric field deforms the liquid meniscus into a conical shape known as a Taylor cone, from which a fine jet emerges and breaks into charged droplets [138]. These droplets solidify through solvent evaporation or gelation and are collected on a grounded surface or in a coagulation or crosslinking bath [139].

The formation and stability of the Taylor cone are critical for controlled particle generation. As voltage increases, electrostatic forces overcome surface tension, producing a stable cone jet mode that generates a continuous stream of droplets [140]. For collagen formulations, cone stability depends on solution properties such as viscosity, conductivity, and surface tension, as well as flow rate and applied voltage. High viscosity or polymer concentration can lead to fiber formation rather than particle formation, while insufficient conductivity may prevent stable jet formation. Therefore, electrospraying requires careful tuning of solution composition and operating conditions to remain within the particle producing regime [141]. Once the jet is formed, it undergoes elongation and breakup into fine droplets due to electrohydrodynamic instabilities. Because the droplets carry like charges, they repel one another, which reduces agglomeration and improves dispersion compared with mechanically generated droplets. Droplet size is governed by multiple factors, including applied voltage, flow rate, solution properties, nozzle geometry, tip-to-collector distance, and ambient conditions [142,143]. In general, higher voltages promote smaller droplets, whereas higher flow rates increase particle size.

**Figure 6:**
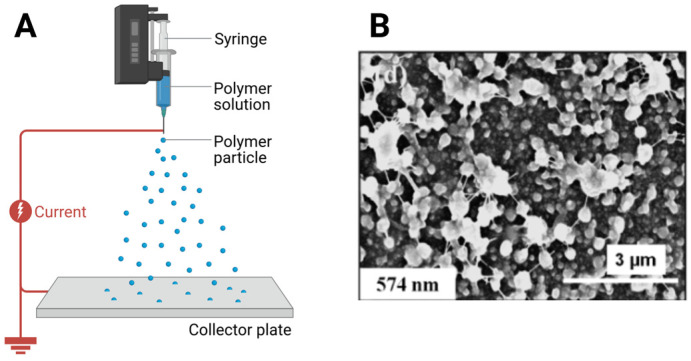
**Electrospraying-based fabrication of collagen microspheres:** Microsphere preparation techniques using electrospraying (**A**) and collagen nanoparticles prepared using electrospraying (**B**) [108].

For collagen systems, this level of control enables the production of particles ranging from submicron to tens of micrometers. For example, collagen solutions (1 percent w/v) in 50 to 80 percent acetic acid have been electrosprayed under conditions such as approximately 25 kV applied voltage, 0.65 mm nozzle diameter, 0.2 mL per minute flow rate, and a 10 cm working distance, with low humidity to promote solvent evaporation [108]. The addition of salts such as NaCl or CaCl_2_ increases solution conductivity and facilitates the formation of quasi monodisperse collagen nanoparticles on the order of 700 to 900 nm, demonstrating the importance of conductivity and molecular structure in controlling particle formation [108]. Figure 6 shows electrospraying-based fabrication of collagen microspheres.

Electrospraying also enables advanced particle architecture. Coaxial configurations allow independent control of core and shell solutions, enabling encapsulation of drugs and spatial separation of components. For instance, collagen theophylline particles have been produced using coaxial electrospraying with controlled flow rates and applied voltage, followed by glutaraldehyde vapor crosslinking to modulate release behavior [107]. Similarly, coaxial electrospraying can be coupled with ionic gelation, where droplets are introduced into a CaCl_2_ bath to form core shell hydrogel structures with tunable dimensions [144]. The technique is also well suited for composite microspheres. Collagen can be combined with synthetic polymers such as PLGA to produce hybrid systems with enhanced mechanical and release properties. In such systems, increasing polymer concentration can shift morphology from particles to fibers due to increased chain entanglement, highlighting the importance of formulation control. Under optimized conditions, spherical microspheres with controlled size distributions can be achieved [37,145]. For example, collagen PLGA composite microspheres loaded with vancomycin have demonstrated sustained release and long-term degradation, illustrating the potential of electrospraying for wound healing and drug delivery applications. After droplet formation, solvent evaporation during flight leads to particle solidification. The final morphology depends on evaporation kinetics and solution composition. In some cases, particles are collected directly on solid substrates, while in others, they are deposited into coagulation or crosslinking baths for additional stabilization. For collagen systems, post-spray crosslinking or fibrillogenesis is often required to enhance structural integrity and control degradation.

Electrospraying offers the advantage of producing fine particles under relatively mild conditions, making it suitable for encapsulating sensitive biomolecules. It eliminates the need for oil phases and enables precise control over particle size and architecture. However, the process is highly sensitive to operating conditions, as stable jet formation requires precise control of voltage, flow rate, and solution properties, and is influenced by environmental factors such as humidity and temperature. Scaling to higher production rates can lead to jet instability, particle aggregation, and non-uniform size distribution, which compromise product quality. In addition, high voltage requirements and solvent handling introduce safety concerns and regulatory burdens, increasing infrastructure and compliance costs. Electrospraying also has relatively low throughput compared with bulk methods and is less suitable for cell-laden systems due to potential adverse effects of electric fields on cell viability [146,147]. From an economic perspective, the need for specialized equipment, skilled operation, and extensive process optimization may limit its widespread adoption compared with conventional emulsification techniques.

Overall, electrospraying produces collagen microspheres through a sequence of Taylor cone formation, jet breakup into charged droplets, and particle solidification via solvent evaporation. Its strengths include fine size control, mild processing conditions, and the ability to generate complex architectures such as core shell and composite particles [146,148]. Despite challenges related to scalability and process sensitivity, electrospraying remains a powerful platform for fabricating collagen-based microspheres with precisely controlled structure and function. To provide a structured overview of how fabrication strategies influence microsphere properties and biological performance, a comparative summary is presented in Table 2. This table links key fabrication methods with resulting structural features, tunable parameters, and corresponding biological outcomes, highlighting the interdependence between processing, structure, and function in collagen microsphere systems.

## 4. Crosslinking Strategies and Functional Modification of Collagen Microspheres

Native collagen gels and microspheres are often too soft and degrade too rapidly for many biomedical applications. Therefore, crosslinking and chemical modification are widely used to enhance mechanical strength, control degradation, and introduce functional properties [149,150,151]. Chemical crosslinking using carbodiimide chemistry, particularly EDC and NHS, is one of the most commonly employed strategies [152]. EDC and NHS function as zero-length crosslinking agents, forming covalent amide bonds between carboxyl and amine groups without introducing a spacer within the network. Because the crosslinker is not incorporated into the final structure, this approach enhances mechanical stability while reducing degradation without adding foreign components to the material [153,154]. Crosslinking increases network density, stabilizes the collagen triple helix, and transforms loosely associated fibrils into an interconnected structure with higher stiffness. As a result, Young’s modulus typically increases by two- to three-fold compared to non-crosslinked collagen. In addition, crosslinking reduces enzymatic degradation by limiting collagenase access to cleavage sites, particularly in the telopeptide regions, thereby significantly extending material stability [155].

Glutaraldehyde crosslinking produces highly robust networks but presents significant toxicity concerns if residual aldehyde groups are not fully quenched [156,157]. These residual groups can react with cellular components, leading to cytotoxicity, chronic inflammation, calcification, and long-term implant failure in some applications such as bioprosthetic heart valves [158]. Consequently, safer alternatives have been developed. Genipin and tannic acid are widely studied biocompatible alternatives that provide effective crosslinking while reducing adverse biological responses [159,160]. Genipin, a naturally derived compound, forms covalent linkages with collagen amino groups and produces nontoxic reaction products. It has been shown to reduce inflammatory responses and promote regenerative macrophage polarization, leading to improved tissue integration and reduced fibrosis in vivo [161]. Tannic acid, a plant-derived polyphenol, interacts with collagen through hydrogen bonding and covalent interactions, enhancing mechanical strength and enzymatic resistance while also providing antioxidant and antimicrobial properties [162]. In various in vivo models, tannic acid crosslinked collagen systems have demonstrated improved biocompatibility, reduced inflammation, and enhanced tissue regeneration [163].

Physical crosslinking methods provide an alternative approach that avoids chemical reagents and associated toxicity. Dehydrothermal (DHT) treatment under heat and vacuum promotes intermolecular bonding through condensation and hydrogen interactions, while ultraviolet or visible light irradiation, with or without photoinitiators, can induce covalent bond formation between amino acid side chains [164,165]. Riboflavin–ultraviolet-A (RF-UVA) crosslinking has emerged as a particularly promising strategy, achieving stabilization comparable to glutaraldehyde while maintaining superior biocompatibility and preserving collagen structure under degradative conditions [165,166].

The enhancement of mechanical properties through crosslinking arises from the formation of additional intermolecular covalent and noncovalent bonds that increase network connectivity and restrict molecular mobility [167]. In native collagen, fibrils are primarily held together by weak interactions such as hydrogen bonding and van der Waals forces, which can be easily disrupted under mechanical loading. Crosslinking introduces stable intermolecular linkages between collagen molecules, effectively increasing the number of load bearing junctions within the network [168]. This transformation shifts the material response from an entropy-dominated regime, where deformation occurs through molecular rearrangement and fibril sliding, to an enthalpy-dominated regime, where deformation requires stretching or breaking of covalent bonds. As a result, crosslinked collagen exhibits increased stiffness, higher tensile strength, and improved resistance to creep and viscoelastic deformation [169].

At the microscale, crosslinking also promotes tighter packing of fibrils and reduces interfibrillar slippage, leading to improved structural integrity and load transfer across the network [155]. The increased crosslink density enhances the effective modulus and reduces strain-dependent softening, which is particularly important for maintaining mechanical stability under physiological loading conditions. In addition, crosslinking can stabilize the triple-helical structure of collagen, further contributing to resistance against thermal and mechanical denaturation. Simultaneously, crosslinking plays a critical role in regulating degradation behavior. The increased network density reduces pore size and matrix permeability, thereby limiting the diffusion of enzymes such as collagenases and other proteases into the interior of the material [151]. Moreover, crosslinking can directly modify or shield enzymatic recognition sites, particularly within the telopeptide regions and specific Gly-X-Y sequences that serve as cleavage points for collagen-degrading enzymes. By restricting both enzyme access and substrate availability, crosslinked collagen matrices exhibit significantly slower degradation rates and prolonged structural stability [170].

Dual crosslinking strategies further enhance these effects by combining complementary mechanisms. For example, integrating carbodiimide chemistry with secondary crosslinkers such as aldehydes or polymer-based systems can increase crosslink density, introduce additional functional groups, and create hierarchical network architectures. These combined approaches can simultaneously improve mechanical strength, reduce swelling, and enhance resistance to enzymatic degradation beyond what is achievable with single crosslinking systems. As a result, dual crosslinked collagen materials often exhibit superior durability, controlled degradation profiles, and improved performance in long-term biomedical applications [171,172].

The choice of crosslinking methods also strongly influences long-term biocompatibility and immune response. EDC and NHS systems are generally considered safe because the crosslinker is not retained in the final structure, although modification of acidic residues may reduce cell adhesion in some cases [154]. In contrast, glutaraldehyde is associated with adverse immune responses and long-term material failure, whereas genipin and tannic acid demonstrate improved immunological tolerance and regenerative outcomes [158]. For collagen microspheres, particularly in regenerative applications, natural crosslinkers such as genipin and tannic acid are often preferred due to their balance of mechanical performance and biological compatibility [173].

Beyond crosslinking, collagen can be chemically modified to further tailor its properties. Incorporation of synthetic polymers such as polyethylene glycol, charged groups, polysaccharides, or bioactive peptides enables control over swelling, surface charge, protein binding, and cell signaling [174,175]. In microsphere systems, these modifications can regulate drug loading and release, enable growth factor immobilization, or create core shell architectures with distinct microenvironments. For example, collagen gelatin hydrogels crosslinked with EDC and NHS and reinforced with HAp nanoparticles exhibit increased mechanical stiffness, tunable degradation, and enhanced pro-regenerative macrophage responses [176]. Similarly, PEG (polyethylene glycol)-modified recombinant collagen systems have demonstrated improved crosslinking efficiency, enhanced cell proliferation, and improved tissue regeneration outcomes [30].

Overall, crosslinking and chemical modification are essential strategies for tailoring collagen microspheres to specific biomedical applications [151]. By selecting appropriate crosslinking chemistries and modification approaches, it is possible to balance mechanical strength, degradation rate, bioactivity, and immune response, thereby enabling the design of collagen-based systems that more closely match the functional requirements of native tissues [177].

## 5. Composite Microspheres

Pure collagen microspheres are highly biocompatible and bioactive; however, they often lack sufficient mechanical strength, degrade rapidly under enzymatic conditions, and provide limited control over release profiles [178]. To address these limitations, collagen is frequently combined with natural or synthetic materials to form composite microspheres, which introduce additional functionalities such as enhanced mechanical stability, osteoconductivity, or antibacterial activity [32].

Hydrophilic polysaccharides are commonly used as complementary components. Alginate, for example, undergoes rapid ionic gelation in the presence of calcium ions, and collagen–alginate microspheres exhibit improved mechanical integrity and handling while maintaining cytocompatibility. Their stiffness, porosity, and release behavior can be tuned by adjusting composition and crosslinking conditions [179]. Agarose provides thermos-reversible gelation and structural support, allowing collagen to function primarily as the bioactive component [180]. Chitosan, a cationic polysaccharide, forms electrostatic interactions with collagen, resulting in stronger and more slowly degrading networks with inherent antibacterial properties, making these systems particularly suitable for wound healing and infection-prone applications [17].

For bone and dental applications, HAp is widely incorporated to enhance osteoconductivity and mimic the mineralized ECM [32]. Collagen–HA microspheres can serve as injectable fillers or carriers for osteogenic factors, with the mineral content and distribution influencing both mechanical properties and degradation behavior [181]. Synthetic polyesters such as Poly-L-lactic acid (PLLA) and poly(lactic-co-glycolic acid) (PLGA) are also used in blended or core shell configurations. In these systems, the polyester phase provides mechanical strength and controlled degradation, while collagen improves cell adhesion and reduces inflammatory responses [105,182].

Collagen can also be combined with decellularized ECM or tissue-specific components to better replicate the native biochemical environment of target tissues such as cardiac, neural, or hepatic systems [183]. These composite microspheres can deliver tissue-specific cues that guide cell differentiation and function. Overall, the selection of partner materials and microsphere architecture serves as a critical design strategy for tailoring collagen-based microspheres to specific biomedical applications [184,185].

## 6. Characterization of Collagen Microspheres

Comprehensive characterization is essential to ensure that collagen-based microspheres possess the physical, chemical, and biological properties required for their intended applications. Small variations in fabrication or crosslinking can significantly influence size, morphology, mechanical behavior, degradation, and cell interactions. Therefore, a combination of complementary analytical techniques is typically employed to establish structure–property–function relationships [50].

### 6.1. Physical Properties

Physical characterization focuses on microsphere size, morphology, surface topography, internal porosity, and mechanical behavior. Particle size and size distribution are commonly measured using optical microscopy with image analysis, laser diffraction, or dynamic light scattering for smaller particles. Narrow size distributions are critical for achieving reproducible drug release profiles and predictable in vivo biodistribution. Scanning electron microscopy (SEM) provides high-resolution images of surface morphology, shape uniformity, and, after fracture or freeze-drying, internal pore structure [186]. Transmission electron microscopy (TEM) can be used to examine fibrillar organization and the distribution of inorganic phases, such as HAp, within composite microspheres [187].

Porosity and pore size distribution influence swelling, diffusion, and cell infiltration. These parameters can be evaluated using image-based analysis of electron or confocal microscopy images, mercury intrusion porosimetry, or gas adsorption techniques, although the latter are typically applied to dried samples. Swelling behavior is assessed gravimetrically by measuring mass changes in aqueous media over time. Mechanical properties, including compressive modulus, yield strength, and viscoelastic response, are measured using micro-compression testing, rheological analysis, or atomic force microscopy-based indentation [188,189]. Together, these measurements provide insight into microsphere performance under physiological conditions.

### 6.2. Chemical and Structural Properties

Chemical and structural characterization is used to confirm collagen identity, evaluate preservation of the triple-helical structure, quantify crosslinking, and assess stability and degradation behavior. Fourier transform infrared (FTIR) and Raman spectroscopy are commonly used to identify characteristic amide bands and monitor structural changes associated with denaturation or crosslinking [190]. Circular dichroism (CD) and differential scanning calorimetry (DSC) provide information on triple helix content and thermal stability, indicating whether processing has altered the native collagen structure [191].

Crosslinking efficiency is typically assessed using colorimetric assays such as 2,4,6-trinitrobenzene sulfonic acid (TNBS) or ninhydrin to quantify residual free amine groups. A decrease in free amine content generally reflects increased crosslink density, although these results are often interpreted alongside mechanical and degradation data to assess functional outcomes. Enzymatic degradation studies using collagenase or other proteases are commonly performed to evaluate mass loss, swelling, and mechanical changes over time [192]. For drug-loaded microspheres, encapsulation efficiency and release kinetics are critical parameters. These are typically measured using ultraviolet (UV)–visible spectroscopy, high-performance liquid chromatography (HPLC), or related analytical techniques to quantify drug concentration in release media over time, enabling modeling of diffusion or degradation controlled release mechanisms [193].

In composite systems, additional techniques may be required. X-ray diffraction (XRD) and energy-dispersive X-ray spectroscopy (EDX) are used to characterize crystalline phases and elemental composition in mineral-containing microspheres, such as collagen HAp systems [194]. Nuclear magnetic resonance (NMR) can be employed to characterize synthetic polymer components or confirm chemical modification of collagen [195]. Collectively, these methods ensure that composition and structure align with design objectives.

### 6.3. Biological Properties

Biological characterization evaluates interactions between collagen microspheres and cells or tissues, including cytotoxicity, biocompatibility, cell adhesion, and immune responses. In vitro cytotoxicity is typically assessed using assays such as MTT (3-(4,5-dimethylthiazol-2-yl)-2,5-diphenyl tetrazolium bromide) or Alamar Blue, where cells are cultured in contact with microspheres or exposed to their extracts [179]. Live and dead staining and fluorescence imaging provide additional qualitative information on cell viability and morphology. Cell adhesion and spreading can be evaluated using immunofluorescence staining of cytoskeletal components such as actin and focal adhesion proteins, combined with quantitative image analysis [196]. For applications involving cell delivery, further studies examine cell proliferation, differentiation, and function over time. For example, osteogenic differentiation can be assessed through alkaline phosphatase activity, mineral deposition, and gene expression, while neural or chondrogenic applications require different marker sets. 

Hemocompatibility and immunological responses are particularly important for systems intended for in vivo use. These can be evaluated through hemolysis assays, platelet adhesion and activation studies, and cytokine production by immune cells such as macrophages or peripheral blood mononuclear cells [21,197]. In vivo studies provide higher level validation of biocompatibility, degradation, and therapeutic performance. Subcutaneous implantation models are commonly used to assess inflammatory response, fibrous capsule formation, and degradation over time. Disease-specific models, such as bone defects, skin wounds, or myocardial infarction, allow evaluation of functional outcomes in relevant tissues. Histological analysis, immunohistochemistry, and imaging techniques such as micro-computed tomography are used to assess tissue integration, vascularization, and regeneration. Together, these studies confirm that collagen-based microspheres are not only structurally and chemically well-defined but also safe and effective in biological environments [82].

### 6.4. Sterilization Compatibility and Material Integrity Considerations

A critical gap in the characterization of collagen microspheres lies in the evaluation of sterilization methods and their impact on material properties and biological functionality. Although collagen-based microspheres must undergo validated sterilization procedures to meet clinical and regulatory requirements [198,199], commonly used methods, including thermal treatment, chemical sterilization such as ethylene oxide (EtO), and radiation-based approaches, can significantly compromise protein bioactivity and structural integrity. These effects may arise from denaturation, unintended crosslinking, or molecular fragmentation [200]. Photochemical crosslinking has emerged as a promising nonthermal and nontoxic alternative that is compatible with sterilization while preserving collagen’s native bioactivity and physicochemical properties [198,200]. However, systematic evaluation of sterilization compatibility for specific collagen microsphere formulations remains limited in the current literature. To address this gap, characterization protocols should include detailed assessment of material properties before and after sterilization. Such analyses are essential to ensure that the mechanical performance, structural integrity, and biological functionality observed in vitro are retained in the final clinical-grade product. This level of validation is critical for demonstrating that regulatory and good manufacturing practice requirements can be met without compromising therapeutic efficacy.

## 7. Biomedical Applications of Collagen-Based Microspheres: Functional Roles, Design Strategies, and Comparative Insights

**Figure 7:**
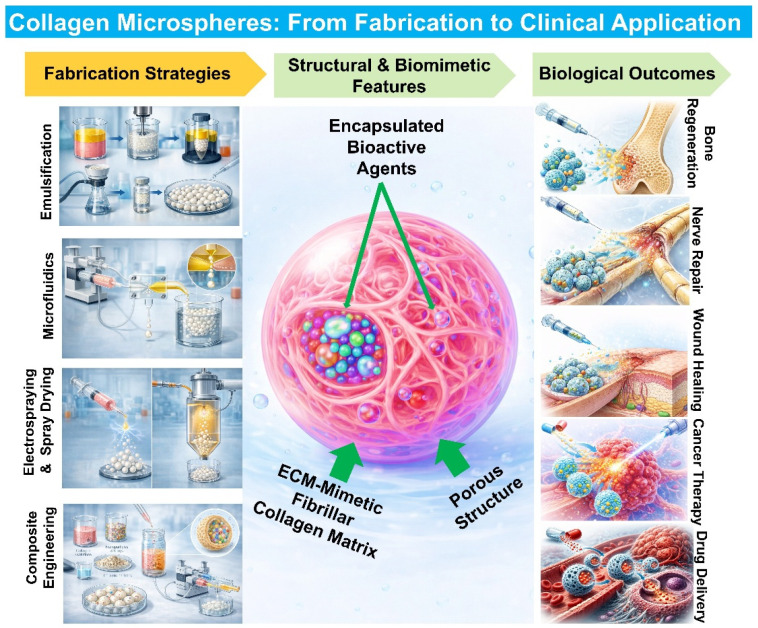
**Overview of collagen microspheres from fabrication to biomedical application**. Various fabrication strategies including emulsification, microfluidics, electrospraying and spray-drying, and composite engineering enable the generation of collagen microspheres with controlled architecture. The microspheres exhibit an ECM mimetic fibrillar collagen matrix, interconnected porous structure, and capacity for encapsulation of bioactive agents. These structural and functional features support diverse biomedical applications, including bone regeneration, nerve repair, wound healing, cancer therapy, and drug delivery. The individual components of this figure were generated using ChatGPT (OpenAI) from a series of prompts (accessed 24 March 2026) and subsequently compiled into a single figure.

Collagen-based microspheres have evolved from simple delivery carriers into multifunctional platforms that integrate structural, mechanical, and biochemical features of native ECM environments. Their injectability, high surface-area-to-volume ratio, intrinsic bioactivity, and modularity collectively distinguish them from traditional scaffold systems and enable their application across diverse biomedical contexts [13,35,201]. A key advantage of collagen microspheres lies in their ability to function across multiple length scales and biological roles. At the microscale, they provide localized niches that regulate cell behavior through matrix-mediated signaling and controlled presentation of therapeutic factors. At the macroscale, they can be assembled into larger constructs or combined with bulk hydrogels to generate structurally complex and spatially heterogeneous tissues. This dual functionality enables flexible therapeutic design and facilitates translation into clinically relevant delivery formats [35,201,202]. The following sections critically examine their performance across major application domains while highlighting design principles and comparative advantages. Figure 7 illustrates an overview of collagen microspheres from fabrication to biomedical application.

### 7.1. Controlled Delivery Platforms: From Molecular Transport to Microenvironment Regulation

#### 7.1.1. Protein and Growth Factor Delivery

Collagen microspheres are widely used for the controlled delivery of growth factors and therapeutic proteins, including bone morphogenetic proteins (BMPs), vascular endothelial growth factor (VEGF), transforming growth factor β (TGF-β), and fibroblast growth factor (FGF) [13,23,35,203]. Unlike conventional bulk hydrogels, where diffusion pathways are long and often poorly defined, microspheres provide reduced and tunable diffusion distances, enabling more predictable and spatially localized release.

Importantly, collagen is not merely a passive carrier. Its molecular structure contains binding domains that interact with growth factors through electrostatic and hydrophobic interactions, improving loading efficiency and retention. This intrinsic affinity distinguishes collagen from many synthetic polymers, which typically require additional functionalization to achieve comparable performance. Furthermore, release kinetics can be modulated through crosslinking density, fibrillar organization, and composite design, allowing simultaneous control over both mechanical stability and molecular transport. For example, genipin crosslinked systems demonstrate that increased crosslink density reduces network permeability and slows release while maintaining bioactivity, resulting in enhanced cellular responses such as osteogenic differentiation [204]. Composite formulations further extend release duration by introducing secondary diffusion barriers, highlighting the importance of multiphase design in achieving clinically relevant delivery profiles [35,200,201].

#### 7.1.2. Small-Molecule and Antimicrobial Delivery

For small-molecule therapeutics, collagen microspheres provide advantages in spatial distribution and sustained release. In contrast to traditional collagen sponges, where drug loading is often achieved by postfabrication soaking and leads to rapid burst release, microspheres enable encapsulation during fabrication, resulting in improved loading efficiency and controlled release behavior. This distinction is particularly important in wound healing, where uniform drug distribution and prolonged exposure are critical. Antibiotic-loaded microspheres have demonstrated sustained release over extended timeframes and improved healing outcomes compared with conventional systems [205]. Additionally, composite microspheres incorporating chitosan introduce antibacterial and hemostatic functionality, illustrating how material selection can be used to integrate multiple therapeutic roles within a single platform [35].

#### 7.1.3. Gene Delivery and Advanced Therapeutic Systems

Collagen microspheres are also being explored as platforms for localized gene delivery, including plasmid DNA, siRNA, and viral vectors. While collagen itself lacks strong cationic character, it can be modified or combined with cationic polymers to facilitate nucleic acid binding and protect genetic cargo from degradation. Beyond simple delivery, these systems enable the creation of localized gene expression niches, where sustained transfection can be achieved while minimizing systemic exposure. However, challenges remain in optimizing transfection efficiency, stability, and scalability. This highlights a broader trend in microsphere design, where balancing biological functionality with translational feasibility remains a central consideration [35,201].

### 7.2. Hard Tissue Applications: Bone Regeneration and Mineralized Microenvironments

Collagen microspheres are particularly well suited for bone regeneration due to their compatibility with mineralized ECM and ability to support osteogenic processes [206,207]. Their function extends beyond passive scaffolding to active participation in tissue regeneration through controlled delivery of morphogens, support of stem cell differentiation, and integration with mineral phases. Accordingly, bone tissue engineering strategies using collagen microspheres generally follow three main approaches: (1) growth factor delivery, where BMP-2- or BMP-7-loaded microspheres act as localized morphogen depots; (2) composite mineralized systems incorporating HAp or β-tricalcium phosphate to enhance osteoconductivity and mechanical performance; and (3) cell-laden microspheres for delivering mesenchymal stem cells to defect sites [35,201].

A key design strategy involves the incorporation of inorganic components such as HAp or β-tricalcium phosphate, which more closely replicates the composite structure of native bone tissue. These mineral phases not only improve mechanical stability but also provide biochemical cues that promote osteogenic differentiation. For instance, the incorporation of HAp nanoparticles into collagen microspheres enables better mimicry of native bone ECM, which contains approximately 65% mineral content, while enhancing mineral deposition and bone ingrowth [206,208]. Representative studies further demonstrate the advantages of such composite systems. Collagen–HAp microspheres containing 10–20 wt% nano β-tricalcium phosphate have shown enhanced porosity (84–94 nm), rapid swelling behavior, high degradation resistance (>80% residual mass after 90 days in collagenase), and sustained calcium release [176]. These properties collectively contribute to improved osteogenic differentiation of pre-osteoblasts and highlight their potential for bone defect repair. In addition, injectable BMP-2-loaded collagen microspheres have demonstrated strong potential as localized morphogen delivery systems, where therapeutic outcomes can be precisely tuned by adjusting dosage and microsphere degradation kinetics [35,201].

Importantly, the injectable nature of collagen microspheres enables minimally invasive delivery to irregular defect sites, addressing key limitations associated with pre-formed scaffolds. However, achieving an optimal balance between mechanical integrity and degradation kinetics remains a critical design consideration for successful clinical translation.

### 7.3. Soft Tissue and Interface Engineering: Cartilage, Tendon, and Complex Tissues

In soft tissue applications, collagen microspheres function as injectable microenvironments that support cell survival, proliferation, and differentiation. Their ability to encapsulate cells within a three-dimensional matrix provides protection during delivery and promotes tissue-specific matrix deposition. For cartilage regeneration, microspheres can be engineered to deliver chondrogenic factors TGF-β, IGF-1 (insulin-like growth factor 1) while maintaining a hydrated and mechanically compliant environment to guide chondrogenesis [34,153]. When combined with bulk hydrogels or polymer networks, they contribute to improved load distribution and structural organization, which are essential for functional tissue regeneration [147].

More broadly, microsphere systems enable bottom-up tissue engineering approaches, where individual microspheres with distinct compositions or functions are assembled to create spatially heterogeneous constructs. This capability is particularly relevant for complex interfaces such as osteochondral or tendon-to-bone regions, where gradients in composition and mechanics are required.

### 7.4. Wound Healing and Skin Regeneration: From Passive Dressings to Active Therapeutic Systems

Collagen-based materials are well established in wound care, but microspheres extend their functionality by transforming passive scaffolds into active therapeutic systems. Their particulate nature allows integration into injectable formulations, sprayable systems, and advanced dressings, enabling more uniform coverage and controlled delivery of therapeutic agents. A key advantage of microspheres is their ability to synchronize degradation with the wound healing process. By tuning crosslinking and particle size, degradation can be aligned with different healing phases, ensuring that the material provides support when needed and is gradually replaced by native tissue.

In addition, microspheres enable multifunctional designs that combine antimicrobial, anti-inflammatory, and pro-regenerative signals. This integrated approach is difficult to achieve with traditional collagen formats and represents a significant advancement in wound management strategies [209,210,211].

### 7.5. Emerging Applications: Neural Repair and Targeted Cancer Therapy

Emerging applications of collagen microspheres include neural tissue repair and localized cancer therapy [35,201,211]. In neural systems, microspheres provide supportive microenvironments that promote axonal growth and cell differentiation, particularly when combined with neurotrophic factors. The modularity of microsphere systems enables incorporation of multiple signaling molecules to create spatially complex neural microenvironments that guide axonal growth and cell migration, critical for peripheral nerve repair. In oncology, collagen microspheres are being investigated as localized drug delivery systems that enhance therapeutic concentration at tumor sites while reducing systemic toxicity. Their degradability and tunable release behavior allow adaptation to tumor microenvironments, although careful design is required to avoid unintended effects on surrounding tissues [35]. Advanced strategies incorporating targeting ligands, stimuli-responsive behavior, and combination therapies highlight the expanding potential of these systems. However, translation to clinical use will require further validation of safety, efficacy, and manufacturability [35].

## 8. Comparative Performance and Translational Considerations of Collagen Microsphere Systems

All three collagen-based formats, including microspheres, sponges, and films, originate from natural collagen and therefore exhibit excellent biocompatibility with minimal inherent immunogenicity [210]. However, differences in structure and processing significantly influence cellular interactions and immune responses. Native collagen scaffolds, particularly sponges that retain fibrillar architecture, generally promote superior cell adhesion and migration compared to highly crosslinked films, which may present a less biologically active surface [212,213]. In this context, collagen microspheres, especially those fabricated with minimal crosslinking or using biocompatible agents such as genipin, can preserve ECM mimetic bioactivity while offering additional design flexibility. Notably, in vivo comparisons between native collagen sponges and EDC crosslinked films have shown improved cell migration and reduced myofibroblast differentiation in native systems, suggesting that microsphere formulations that retain native collagen features may provide favorable immunological outcomes [212].

A major advantage of collagen microspheres lies in their injectability and compatibility with minimally invasive delivery. Microspheres, typically ranging from 2 to 500 μm in diameter, can be administered through standard clinical needles (18–27G), enabling percutaneous delivery to irregular defect sites without the need for open surgery [35,201]. In contrast, collagen sponges and films require surgical placement, limiting their clinical flexibility. This injectable capability reduces surgical trauma, shortens operative time, and lowers infection risk, while also improving patient recovery [214]. In wound healing applications, microspheres can be incorporated into injectable hydrogels or applied as sprayable formulations, whereas sponges and films require manual placement and alignment [35,201,215]. This level of control is particularly advantageous in regenerative applications, where scaffold degradation can be synchronized with specific healing phases, including inflammation, proliferation, and remodeling.

From a manufacturing perspective, sponges and films benefit from relatively simple and well-established fabrication methods such as lyophilization and solvent casting, making them cost-effective and scalable [210,215]. In contrast, microsphere fabrication techniques, including emulsification, microfluidics, and electrospraying, are more complex and may increase production cost and variability [201]. However, this added complexity enables precise control over microsphere properties such as size, crosslinking density, and payload distribution, which is critical for advanced therapeutic applications requiring controlled delivery and spatial precision.

In terms of clinical translation, collagen sponges remain the most widely adopted format, with multiple Food and Drug Administration (FDA)-approved products such as Integra™ and Avance™ used in wound healing and bone regeneration [203]. Collagen films are also gaining traction, particularly as barrier membranes in periodontal and guided tissue regeneration applications. In contrast, collagen microspheres, despite their clear functional advantages, are largely in preclinical or early clinical stages. This reflects both the relative novelty of the platform and regulatory pathways that favor established material formats. Continued advances in scalable manufacturing, reproducibility, and regulatory validation will be essential for broader clinical adoption.

## 9. Advanced Biofabrication and Emerging Translational Applications

Advanced biofabrication strategies are rapidly expanding the translational potential of collagen microspheres by enabling precise spatial organization, patient-specific design, and integration with emerging manufacturing technologies. Among these approaches, three-dimensional bioprinting has emerged as a particularly powerful platform for constructing anatomically complex and functionally graded tissues. Collagen-based bioinks, typically formulated at 2–6 mg mL^−1^, are widely used due to their inherent biocompatibility, cell-adhesive properties, and enzymatic degradability, which support dynamic tissue remodeling [216,217,218]. The incorporation of collagen microspheres into these bioinks introduces a bottom-up fabrication paradigm, where microspheres act as modular building units that can be spatially organized within printed constructs to generate heterogeneous microenvironments. This strategy enables precise control over cell distribution, matrix composition, and localized delivery of bioactive cues, allowing the fabrication of constructs that more closely replicate the structural and functional complexity of native tissues [35,201].

Despite these advantages, several technical challenges must be addressed to enable clinical translation. Collagen exhibits strong temperature- and pH-dependent fibrillogenesis, requiring strict control of printing conditions to prevent premature gelation. Bioinks are typically maintained at low temperature prior to printing and neutralized during or after deposition, necessitating specialized printing systems with temperature control [216,217]. In addition, extrusion-based bioprinting introduces shear stresses that can compromise cell viability, often resulting in postprinting viability between 50% and 85%. Optimization of extrusion parameters, nozzle geometry, and incorporation of protective polymers can improve cell survival while maintaining print fidelity [216]. Tradeoffs between resolution and throughput also remain a critical limitation, as higher-resolution techniques provide finer structural detail but reduce fabrication speed. Furthermore, printed constructs require postfabrication maturation in bioreactors over several weeks to achieve functional tissue properties through mechanical stimulation, biochemical conditioning, and vascularization support [216,217].

Beyond biofabrication, collagen microspheres enable advanced control over localized cellular microenvironments, which is essential for next-generation regenerative therapies [118]. Their modular structure allows integration of multiple signaling modalities within a single system, enabling spatially and temporally controlled presentation of biochemical cues. This capability is particularly relevant for complex tissues that require coordinated signaling gradients or staged therapeutic delivery [118]. In gene delivery applications, collagen microspheres have been explored as localized platforms for presenting nucleic acids, including plasmid DNA and siRNA [219]. Although collagen is not inherently cationic, it can be modified or combined with cationic polymers to enhance nucleic acid binding and cellular uptake, enabling sustained and localized transfection while minimizing systemic exposure. However, achieving optimal transfection efficiency, stability, and biosafety remains an ongoing challenge.

In translational contexts such as wound healing, collagen microspheres support the development of dynamic therapeutic systems that extend beyond conventional passive materials. Their particulate nature enables incorporation into injectable formulations, sprayable systems, and composite hydrogels, allowing improved coverage of irregular defects and localized delivery of therapeutics [205,213]. Importantly, their degradation behavior can be precisely tuned through crosslinking density and particle size, enabling synchronization with the temporal progression of tissue repair. Emerging strategies include sequential or staged release systems, where multiple therapeutic agents are delivered in a controlled temporal sequence to regulate inflammation, promote tissue formation, and reduce scarring [220]. Such approaches highlight the potential of microsphere-based systems to actively guide the healing process rather than simply provide structural support.

Collagen microspheres are also being explored in emerging applications such as localized cancer therapy, where their ability to deliver therapeutics directly to tumor sites offers significant advantages over systemic administration. Microsphere-based depots can be engineered to provide sustained and localized drug release, increasing intratumoral drug concentration while minimizing systemic toxicity [221]. In addition, the enzymatic degradability of collagen may enable responsiveness to tumor-associated protease activity, allowing environment-sensitive release profiles. However, this also requires careful control to avoid premature drug release or unintended damage to surrounding healthy tissues. Advanced strategies incorporating targeting ligands, stimuli-responsive elements such as pH or enzyme sensitivity, and combination therapies including chemo-immunotherapy are being investigated to enhance therapeutic specificity and efficacy while maintaining safety and manufacturability [221,222].

Overall, advanced biofabrication approaches and emerging translational applications position collagen microspheres as a highly adaptable platform for next-generation regenerative and therapeutic systems. Their ability to integrate precise spatial control, dynamic microenvironment regulation, and minimally invasive delivery provides significant advantages over traditional biomaterial systems. However, challenges related to scalability, reproducibility, and regulatory validation remain key barriers to clinical translation, underscoring the need for continued development of standardized manufacturing and characterization strategies.

## 10. Preclinical Evidence, Translational Landscape, and Clinical Outlook of Collagen Microspheres

Collagen microspheres have demonstrated strong preclinical performance across diverse biomedical applications, including bone regeneration, neural repair, and oncology. These studies collectively highlight the ability of collagen to function as an active biological component that enhances therapeutic outcomes through improved cell–material interactions, controlled delivery of bioactive agents, and formation of biomimetic microenvironments [223,224,225,226]. In bone regeneration, collagen-based composite microspheres have been successfully integrated with mineral phases and growth factors to promote osteogenesis. Seong et al. (2020) developed biphasic calcium phosphate–collagen microspheres for sustained delivery of bone morphogenetic protein 2 (BMP-2), demonstrating enhanced drug retention and superior bone regeneration in a rabbit defect model compared with uncoated controls [223].

Similarly, Zhang et al. (2022) engineered PLLA–collagen–nano HAp microspheres with a hierarchical “pomegranate” structure, where collagen serves as the primary matrix while synthetic and mineral components provide mechanical strength and osteoconductivity [224]. These microspheres exhibited excellent biocompatibility, with cell viability exceeding 100 percent, controlled degradation over approximately 60 days, and significant upregulation of osteogenic markers. Their mesoporous architecture further supported nutrient transport and cell migration, underscoring the importance of composite design in optimizing bone tissue regeneration.

Beyond skeletal applications, collagen microspheres have shown strong potential in neural tissue engineering. Zolfagharzadeh et al. (2022) developed collagen–hyaluronic acid (HA) composite microspheres using enzymatic crosslinking within a microfluidic platform, enabling fabrication without toxic reagents while preserving collagen bioactivity [225]. In a rat sciatic nerve injury model, these microspheres significantly improved axonal regeneration, remyelination, and functional recovery, as evidenced by enhanced sciatic functional index values, improved sensory response, and increased muscle regeneration. Histological analyses further confirmed improved nerve organization and myelin structure, demonstrating the ability of collagen-based systems to support complex neural repair processes.

Collagen microspheres have also been extended to oncology applications, highlighting their versatility beyond regenerative medicine. Li et al. (2025) developed polyvinyl alcohol–collagen microspheres labeled with iodine-131 for targeted radioembolization of hepatocellular carcinoma [226]. These microspheres achieved high radiolabeling efficiency and demonstrated precise tumor localization with sustained retention over 14 days, corresponding to the radioisotope half-life. Treated animals showed significant tumor growth inhibition and improved survival, establishing collagen microspheres as promising carriers for localized cancer therapy.

The growing body of intellectual property surrounding collagen microspheres further underscores their strong translational potential. Early patents established foundational technologies in collagen crosslinking and hydrogel systems, while more recent developments have focused on functionalized collagen, composite formulations, and stimuli-responsive platforms. These innovations span a wide range of fabrication approaches, including emulsification, crosslinking, and lyophilization, as well as diverse biomedical applications such as drug delivery, tissue engineering, and injectable therapeutics. A representative summary of key patents, including fabrication strategies, material innovations, and application areas, is provided in Table 3. The global distribution of these patents across major jurisdictions highlights increasing commercial interest and reflects the maturation of collagen microsphere technologies toward clinical translation.

Despite strong preclinical validation, clinical translation of collagen microspheres remains limited, with no reported human clinical trials to date. This gap reflects common challenges associated with advanced biomaterials rather than limitations in efficacy. One of the primary barriers is manufacturing scale-up. While current fabrication methods are well suited for laboratory-scale production, clinical translation requires large-scale, reproducible manufacturing under good manufacturing practice conditions. Composite microspheres introduce additional complexity, including the need for uniform distribution of multiple components and rigorous quality control. Variability in collagen source and processing further complicates standardization, necessitating controlled sourcing strategies or development of recombinant collagen systems.

Regulatory considerations also present significant challenges. Depending on their composition and intended application, collagen microspheres are often classified as combination products, requiring compliance with both device and drug regulatory pathways. Although existing preclinical studies provide strong evidence of efficacy and short-term biocompatibility, additional long-term safety, immunogenicity, and pharmacokinetic studies are required to meet regulatory standards.

Long-term biocompatibility and degradation behavior remain critical areas for further investigation. Most current studies evaluate outcomes over relatively short durations, whereas clinical applications require extended assessment to ensure sustained performance, safe degradation, and absence of adverse immune responses. Particular attention must be given to the immunogenic potential of animal-derived collagen and the safety of degradation products from composite systems.

Economic considerations also play a key role in limiting clinical translation. The cost of early phase clinical trials, combined with the complexity of regulatory approval, represents a significant barrier for academic and early-stage ventures. However, funding mechanisms such as SBIR programs, foundation grants, and industry partnerships provide viable pathways to support translational development. Advances in clinical trial design and strategic integration with existing approved therapies may further accelerate clinical adoption.

Overall, the convergence of strong preclinical evidence, expanding intellectual property, and advancing manufacturing technologies positions collagen microspheres as a highly promising platform for next-generation regenerative medicine and targeted therapeutic delivery. Continued efforts in standardization, scalability, and regulatory validation will be essential to bridge the gap between laboratory success and clinical implementation.

## 11. Challenges and Future Perspectives

Despite their significant promise, several fundamental challenges must be addressed before collagen microspheres can achieve broad and reliable clinical translation. One of the most critical barriers is scalable and reproducible manufacturing. While conventional emulsification methods are readily scalable, they often produce polydisperse microspheres with limited control over size, porosity, and internal architecture. In contrast, microfluidic systems offer excellent precision and monodispersity but remain constrained by low throughput and scalability challenges [96,201]. Emerging approaches such as electrospraying and spray-drying provide alternative routes for large-scale production; however, these methods require further optimization to preserve collagen’s native structure, maintain bioactivity, and ensure batch-to-batch reproducibility [181,231]. Beyond fabrication, achieving consistent collagen quality, controlled crosslinking density, endotoxin removal, and robust quality assurance remains essential, particularly for microspheres incorporating biologics.

Material variability and safety considerations represent another major translational challenge. Collagen derived from animal sources exhibits inherent batch-to-batch variability in molecular composition, crosslinking behavior, and potential immunogenic epitopes. Although purification strategies and the use of atelocollagen can reduce antigenicity, complete elimination of immune response risks remains difficult. Recombinant and engineered collagen systems offer promising alternatives with improved consistency and tunability, but their large-scale production and cost-effectiveness require further development [214,232,233]. In parallel, the selection of crosslinking chemistries must balance mechanical stability with cytocompatibility, as residual crosslinking agents or excessive network stiffness can negatively impact cellular responses.

Sterilization and regulatory compliance introduce additional layers of complexity. Standard sterilization methods, including thermal, chemical, and radiation-based approaches, can compromise collagen structure and bioactivity, making it challenging to maintain functional integrity in clinical-grade products. Consequently, there is a need for sterilization strategies that preserve both mechanical and biological properties while meeting regulatory standards. Furthermore, collagen microspheres are often classified as combination products when loaded with therapeutic agents, requiring coordinated regulatory pathways that integrate device, drug, and biologic considerations. This significantly increases the burden of preclinical validation, including long-term safety, immunogenicity, degradation profiling, and pharmacokinetics under physiologically relevant conditions [234,235,236].

Looking forward, the future of collagen microspheres lies in their integration with advanced biofabrication and precision medicine strategies. The convergence of microsphere technologies with 3D bioprinting will enable the fabrication of spatially organized, patient-specific constructs, where microspheres function as modular building blocks or bioactive components within hybrid bioinks [237]. In parallel, the development of stimuli-responsive systems incorporating enzyme-sensitive, pH-responsive, or redox-responsive mechanisms will allow dynamic and on-demand therapeutic release tailored to evolving disease environments [238]. Such systems have the potential to move beyond passive delivery toward adaptive and feedback-driven therapeutic platforms.

Multifunctional microspheres capable of sequential or combinatorial delivery of growth factors, nucleic acids, and immunomodulatory agents represent another important direction. These systems aim to better replicate the temporal complexity of native healing cascades, enabling coordinated regulation of inflammation, tissue formation, and remodeling. Advances in engineered and recombinant collagen will further enhance this capability by allowing precise incorporation of bioactive motifs, improved control over degradation, and reduced immunogenicity [239].

Additional opportunities include integration with stem cell therapies to enhance cell survival, retention, and lineage specific differentiation, as well as the development of minimally invasive injectable systems for targeted and localized treatment. The incorporation of imaging agents into microspheres may enable real-time monitoring of biodistribution, degradation, and therapeutic response, supporting more effective and personalized treatment strategies. Moreover, the application of computational modeling and artificial intelligence to microsphere design, including optimization of size distribution, release kinetics, and structural architecture, has the potential to significantly accelerate development cycles and improve predictive performance.

Ultimately, successful clinical translation will depend on the establishment of standardized protocols for fabrication, characterization, and reporting. It also depends on robust manufacturing processes that ensure reproducibility and scalability. Addressing these challenges is essential to realize the full potential of collagen microspheres as next-generation platforms for regenerative medicine and targeted therapeutic delivery.

## 12. Conclusions

Collagen-based microspheres represent a versatile and clinically relevant biomaterial platform that integrates the biological functionality of native ECM with the engineering advantages of modular and injectable systems. Through careful control of collagen source, crosslinking chemistry, composite design, and fabrication strategy, these microspheres can be tailored to achieve controlled therapeutic release, support cell delivery, and promote tissue regeneration across a wide range of applications, including drug delivery, bone and cartilage repair, wound healing, and localized cancer therapy. Despite these advances, several critical challenges must be addressed to enable successful clinical translation. These include the development of scalable and reproducible manufacturing processes, implementation of sterilization methods that preserve bioactivity, mitigation of immunogenicity risks associated with collagen sources, and establishment of consistent clinical-grade quality standards. Looking forward, continued progress in areas such as 3D biofabrication, stimuli-responsive systems, multifunctional and sequential delivery strategies, and recombinant or engineered collagen will further enhance the functionality and reliability of these systems. Addressing these challenges in parallel with technological innovation will be essential to transition collagen microspheres from promising research platforms to robust and widely adopted clinical therapies.

## Figures and Tables

**Table 1:** Summary of representative fabrication techniques for collagen-based microspheres.

Techniques	Principle (Very Brief)	Size Control	Key Advantages	Limitations/Notes
Emulsification	Collagen droplets formed in oil phase and gelled by pH, temperature, or crosslinking	Broad (1 to 1000 µm)	Simple; scalable; versatile payload loading	Polydispersity; oil/shear effects [13,23,92,93,94]
Microfluidics	Droplet generation in microchannels (T-junction/flow-focusing)	Highly monodisperse	Precise size; cell-level control	Low throughput; scale-up required [95,96,97,98,99]
Spray-drying	Atomization into heated gas with rapid solvent evaporation	Moderate	Continuous; dry, storage-stable	Thermal denaturation risk [100,101,102,103,104]
Electrospraying	Electrohydrodynamic droplet formation under high voltage	Fine (0.1 to 10 µm)	No oil; low thermal stress; core–shell	Limited cell compatibility [105,106,107,108]

**Table 2:** Relationship between fabrication strategy, structural features, and biological outcomes in collagen microspheres.

Fabrication Strategy	Key Structural Features	Tunable Parameters	Biological Outcomes
Water-in-oil (W/O) emulsification	Polydisperse microspheres; porous or dense structure depending on crosslinking	Stirring speed, surfactant concentration, crosslinking density	Moderate control over release; supports cell adhesion; batch variability may affect reproducibility
Microfluidics	Highly monodisperse size; uniform architecture; controlled internal structure	Flow rate ratios, channel geometry, gelation kinetics	Precise and reproducible drug release; uniform cell response; improved predictability in vivo
Electrospraying	Fine droplets; relatively uniform size; potential nano/micro-scale control	Voltage, flow rate, nozzle distance	High surface area enhances release kinetics; may affect protein integrity if not optimized
Spray-drying	Dense or hollow particles; rapid solvent removal; scalable	Temperature, feed rate, solvent system	Fast production; potential loss of bioactivity; useful for stable formulations
Crosslinker-free fibrillization	Native-like fibrillar structure; high ECM mimicry	Temperature, pH, fibrillization time	Preserves bioactivity; promotes cell adhesion and remodeling; faster degradation
Composite microspheres (e.g., collagen + HA, alginate, nHA)	Multiphase structure; enhanced stiffness and biofunctionality	Composition ratio, crosslinking type, mineral content	Improved mechanical strength, osteoconductivity, antibacterial effects; tailored degradation
Double emulsion (W/O/W)	Core–shell structures; encapsulated inner phase	Inner/outer phase composition, surfactants	Controlled and sustained release of sensitive payloads; reduced burst release

**Table 3:** Representative patents on collagen-based microsphere fabrication strategies and biomedical applications.

Patent #	Title	Year	Assignee	Microsphere Type	Key Innovation/Findings	Status
US7931918B2 [227]	Collagen-based microspheres and methods of preparation and uses thereof	2005–2011	University of Hong Kong	Collagen microspheres (photochemically crosslinked)	High encapsulation efficiency, sustained protein release, tunable mesh size via photosensitizer dosage	Expired (2025). Public Domain
EP2175978B1 [227]	Collagen-based microspheres and methods of preparation and use thereof	2008–2019	International	Collagen microspheres	Improved drug loading, sustained release, biocompatible formulations	Granted & Active
EP1707260A1 [92]	Method of preparing crosslinked collagen microspheres	2003–2006	Patent Cooperation Treaty	Crosslinked collagen microspheres	Large surface-area-to-volume ratios, scalable manufacture, enhanced regeneration properties	Published
US20250161539 [228]	Injection of collagen elastin hydrogel microparticles into torn tendons and ligaments	2023–2025	Private Patent	Collagen–elastin composite microparticles	Injectable formulation, minimally invasive delivery, protein biocoacervate technology	Recently Issued (May 2025)
CN112755924A [229]	Preparation method of vinyl collagen microspheres	2021	Chinese Institution	Vinyl-modified collagen microspheres	Enhanced chemical modification for improved properties and bioactivity	Published
CN113926435A [230]	Preparation method and application of collagen microsphere adsorbent	2021–2022	Huazhong University of Science and Technology	Collagen microspheres (adsorbent application)	Porous structure, high adsorption capacity, biocompatible formulation	Published

## Data Availability

No new data were created or analyzed in this work. Data sharing is not applicable to this article.
